# Intestinal microbiota analysis and network pharmacology reveal the mechanism by which Lianhua Qingwen capsule improves the immune function of mice infected with influenza A virus

**DOI:** 10.3389/fmicb.2022.1035941

**Published:** 2022-11-25

**Authors:** Ping Xu, Zhu Yang, Shuangqiu Du, Zongyuan Hong, Shuzhi Zhong

**Affiliations:** ^1^Wannan Medical College, Wuhu, China; ^2^Nanjing University of Chinese Medicine, Nanjing, China

**Keywords:** influenza A virus, intestinal microbiota, network pharmacology, TLR4/NF-κB signalling pathway, Lianhua Qingwen capsule

## Abstract

**Objective:**

Lianhua Qingwen capsule (LHQW) can attenuate lung injury caused by influenza virus infection. However, it is unclear whether the intestinal microbiota plays a role in LHQW activity in ameliorating viral infectious pneumonia. This study aimed to investigate the role of intestinal microbiota in LHQW activity in ameliorating viral infectious pneumonia and its possible mechanisms.

**Research design and methods:**

A mouse model of influenza A viral pneumonia was established by intranasal administration in BALB/c mice. Detection of influenza virus in the lungs, pathological examination of the lungs and small intestine, and biochemical detection of inflammatory indices were performed. The effects of LHQW on intestinal microbiota were evaluated by 16S rRNA gene sequencing. The key components and targets of LHQW were screened *via* network pharmacology and verified through molecular docking, molecular dynamics simulation, and free binding energy calculations.

**Results:**

Body weight decreased, inflammatory factor levels were disturbed, and the lung and intestinal mucosal barriers were significantly injured in the infected group. The alpha diversity of the intestinal microbiota decreased, and the abundance of *Bacteroidetes, Muribaculaceae*_unclassified, and *Streptococcus* decreased significantly. LHQW treatment reduced the viral load in the lungs, rescued body weight and survival, alleviated lung and intestinal mucosal barrier injury, reversed the reduction in the intestinal microbiota alpha diversity, and significantly increased the abundance of *Bacteroidetes* and *Muribaculaceae*. Network pharmacological analysis showed that six active herbal medicinal compounds from LHQW could regulate the intestinal microbiota and inhibit the immune-inflammatory response through the Toll-like receptor (TLR) and nuclear factor-κB (NF-κB) signalling pathways in the lungs.

**Conclusion:**

These results suggest that LHQW is effective for treating influenza A virus infectious pneumonia, and the mechanism is associated with the regulation of the TLR4/NF-κB signalling pathway in the lungs by restoring intestinal microbiota and repairing the intestinal wall.

## Introduction

In the past two decades, there have been several respiratory virus pandemics, which seriously threaten human health and negatively impact on the national economy and social life worldwide. Influenza viruses circulating in humans are mainly of two subtypes: type A (HA), H1N1, and H3N2. Human seasonal influenza virus A can generate new strains *via* antigenic drift. The new strain overcomes existing immunity in humans, leading to a new influenza epidemic (Caton et al., 1982; Treanor, 2004; Boni, 2008). Another significant way of influenza virus evolution leading to pandemics is the antigenic shift, in which the viral genome is rearranged to produce new subtypes with significant antigenic changes (Webster et al., 1982; Zhang et al., 2017). If the new influenza virus spreads efficiently from person to person, it could lead to a global pandemic. Four classes of antiviral drugs are currently approved worldwide for the treatment of influenza: amamantane derivatives, neuraminidase inhibitors, membrane fusion inhibitors, and RNA-dependent RNA polymerase inhibitors (Li et al., 2022). Due to mutations and rearrangements in the influenza virus genome, the antiviral effect of these drugs is also decreasing (Paules and Subbarao, 2017); therefore, it is necessary to explore new therapeutic strategies.

Lianhua Qingwen capsule (LHQW), a traditional Chinese medicine compound, has complex components and various effects (Liang et al., 2020; Huang et al., 2021; Zhou et al., 2021). The LHQW was first used clinically in 2003. Since then, it has been used to treat atypical pneumonia, severe acute respiratory syndrome coronavirus (SARS-CoV), Middle East respiratory syndrome coronavirus (MERS-CoV), influenza A H1N1 virus, influenza A H3N2 virus, and influenza H7N9 virus infections (Xing and Liu, 2021). In 2020, the LHQW capsule was recommended for COVID-19 treatment in China (Ni et al., 2020; Wu et al., 2020; Zhuang et al., 2020; Li et al., 2021; Hu et al., 2022). Literature and clinical evidence have indicated that LHQW, which ameliorates symptoms of influenza, regulates the expression of cytokines after viral infection, alleviates lung injury, and has protective effects against COVID-19 (Liang et al., 2020; Huang et al., 2021; Shen and Yin, 2021; Hu et al., 2022). LHQW, combined with routine treatment, can be used as synergistic therapy to significantly improve symptoms and reduce the mortality rate of critically ill patients (Wu et al., 2021; Yang et al., 2021). Animal experiments have also confirmed that LHQW can reduce the viral load in the lungs of mice with viral pneumonia, reduce the expression of inflammatory factors in the lungs, and improve lung injury (Xia et al., 2020; Su et al., 2022). It is well known that viral clearance in the lung depends mainly on lymphocytes and macrophages (Bedi et al., 2022; Cammann et al., 2022; Harris and Borg, 2022; McGee et al., 2022; Verma et al., 2022; Wei et al., 2022; Zhang H. et al., 2022; Zhang M. et al., 2022); however, the present findings do not clarify how LHQW reduces the viral load in the lung and improves lung injury with viral pneumonia.

According to TCM theory, the lung and large intestine interact in terms of physiological, pathophysiological, and immune functions. Both belong to the mucosal immune system, although the intestine and respiratory tract are two separate organs. The latest research has indicated that changes in intestinal microbial composition and function are correlated with the development of lung diseases (“intestine–lung axis”) (Bulanda and Wypych, 2022; Chen et al., 2022; Melo-Gonzalez et al., 2022; Shi et al., 2022; Wang L. et al., 2022; Wang Z. et al., 2022). Moreover, available data indicate that intestinal microbes are strongly correlated with clinical symptoms and inflammatory indices of severe acute respiratory syndrome coronavirus 2 (SARS-CoV-2) infection (Wang H. et al., 2021; Gutierrez-Castrellon et al., 2022; Sencio et al., 2022; Xavier-Santos et al., 2022). However, the composition and characteristics of the intestinal microbiota altered by LHQW treatment for influenza A virus infection and the relationship between microbiota changes and the antiviral effects of drugs need to be further explored.

This study aimed to investigate the role of intestinal microbiota in LHQW activity in ameliorating viral infectious pneumonia and its possible mechanisms. In this study, 16S rRNA was used to analyze the characteristics of the intestinal microbiota. Computer-aided design technology was used to integrate disease, drug-related genes, and proteins for comprehensive analysis to explore the relationship between microbes and respiratory diseases mediated by LHQW through the “intestine-lung axis” and the potential molecular mechanism of the major active components and targets of LHQW.

## Materials and methods

### Animal experiment

#### Reagents and materials

A total of 90 SPF BALB/c mice aged between 6 and 8 weeks (half males and half females) were used in this study. Mice were purchased from Qinglongshan Animal Farm, Jiangning District, Nanjing. The same pathogen-free room was used to accommodate all the mice at 18–25°C and 50–60% humidity. All animal experimental procedures strictly followed the protocol approved by the Ethics Committee of Wannan Medical College (YJS-2020-10-006).

Anti-TLR4 rabbit pAb (A11226, ABclonal Technology Co., Ltd., China), anti-NF-κB p65 rabbit mAb (D14E12, Cell Signaling Technology, United States), anti-phospho-NF-κB p65 (Ser536) anti-rabbit mAb (93H1, Cell Signaling Technology, USA), anti-MyD88 rabbit mAb (D80F5, Cell Signaling Technology, USA), anti-occludin rabbit anti-mouse (ab216327, Abcam, United Kingdom), and anti-ZO1 rabbit anti-mouse (ab216880, Abcam, UK) were used as primary antibodies. The secondary antibody was horseradish enzyme-labelled anti-rabbit IgG [Zsbio ZB-2301]. TRNzol Universal Total RNA Extraction Reagent (DP424, Beijing), Scientific Revertaid First-Strand cDNA Synthesis Kit (10,071,325, Thermo Fisher, USA), and One-Step Prime Script RT–PCR Kit (Perfect Real Time; RR064A, Takara, Japan) were used. Primers were synthesized by Shanghai Shenggong Bioengineering Technology Co., Ltd. Lianhua Qingwen capsules (Z20040063, Yiling, China) and oseltamivir phosphate capsules (H20065415, China Kewei) were used. A nucleic acid protein tester (Biophotometer D30, Germany), high-speed freezing centrifuge (Beckman 64R, USA), and real-time fluorescence quantitative PCR instrument (ABI7500, USA) were used. An enzyme-linked immunosorbent assay (ELISA) test kit for Lipopolysaccharide (LPS) was purchased from Hangzhou Lianke Biotechnology Co., Ltd.

#### Grouping and administration

The A/Puerto Rico/8/34 (H1N1; PR8) strain was subcultured in chicken embryos. The titer of the amplified virus was 1:440, and the median lethal dose (LD50) was 10^–2.1^/50 μl. After the mice were lightly anesthetized with ether, 50 μl of 10^–2.1^ LD50 influenza virus solution was evenly dropped into the nostrils of each mouse to establish the model. The control group(control) received nasal drip of normal saline. Mice were randomly assigned to a control group (18) or an infected group (Buckley and Turner, 2018; Zhang et al., 2021). The mice were fed normally after the nasal drip.

**Figure 1:**
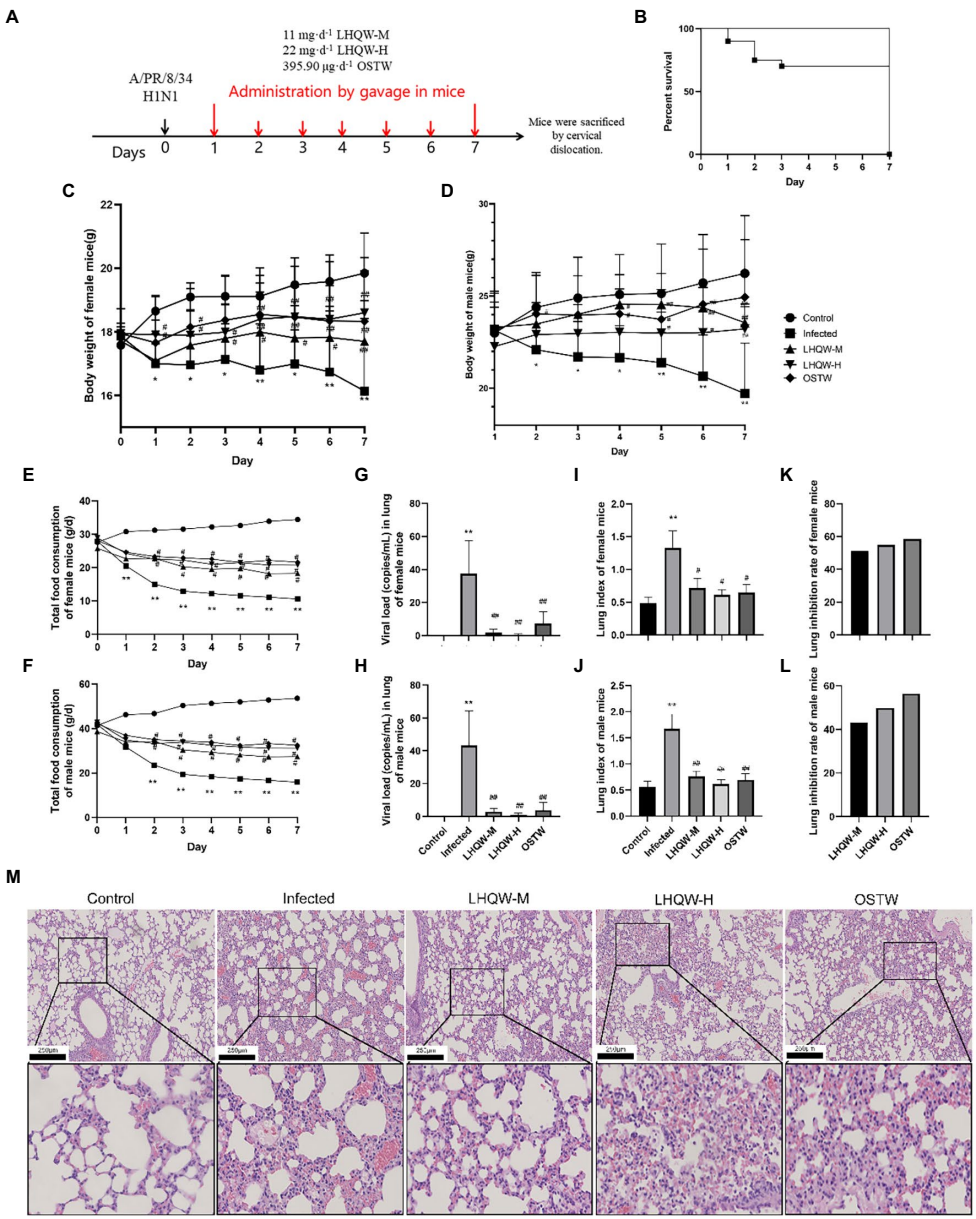
LHQW improves lung injury and inflammation. **(A)** Flow diagram; **(B)** Mouse survival curve: The survival rate of mice in other groups except the infected group (72%) were 100%(*n* = 90); **(C)** Weight changes of female mice infected with influenza virus **(G)** (*n* = 45); **(D)** Weight changes of male mice infected with influenza virus (g; *n* = 45); **(E)** Daily changes of total food consumption of female mice in each group (g/d; *n* = 45); **(F)** Daily changes of total food consumption of male mice in each group (g/d; *n* = 45); **(G)** Viral load (copies/mL) in lung tissue of female mice (*n* = 45); **(H)** Viral load (copies/mL) in lung tissue of male mice (*n* = 45); **(I)** Lung index of female mice (*n* = 45); **(J)** Lung index of male mice (*n* = 45); **(K)** Lung inhibition rate of female mice (*n* = 45); **(L)** Lung inhibition rate in male mice (*n* = 45); **(M)** HE staining of mouse lung tissue (200 and 400 times). The mice were randomly assigned to the virus infected group (infected), 11 mg·d^−1^ (LHQW-M) Lianhua Qingwen capsule, 22 mg·d^−1^ (LHQW-H) Lianhua Qingwen capsule high-dose group and 395.90 μg·d^−1^ oseltamivir group (OSTW). Compared with control group, ^*^p < 0.05, ^**^p < 0.01; Compared with infected group, ^#^*p* < 0.05, ^##^*p* < 0.01.

The incidence and death of mice were recorded daily. After nasal drip, the mice were randomly assigned to the virus-infected group (infected), 11 mg d^−1^ (LHQW-M) Lianhua Qingwen capsule, 22 mg d^−1^ (LHQW-H) Lianhua Qingwen capsule high-dose group and 395.90 μg d^−1^ oseltamivir group (OSTW) (Figure 1A). There were 9 female and 9 male mice in each group. The drug group was administered 0.2 ml by gavage at the same time for 7 days, whereas the control and infected groups were given the same amount of distilled water by gavage. The gavage volume of mice is 0.1–0.2 ml/10 g, and what we used is 0.2 ml/20 g. Firstly, opened the Lianhua Qingwen capsule, accurately weighed the powder in the capsule, added it to distilled water, and prepared solutions with different final concentrations. Then, stored the prepared solution in a refrigerator at 4°C, and recovered to room temperature half an hour before each gavage. Food consumption was calculated by weighing the added feed with an electronic balance on the same day and the remaining feed on the next day. The total food intake of the cage mice was the amount of feed added minus the remaining amount (Total food intake of each group of mice).

#### Sample collection and preparation

Mice were anesthetized with ether. Blood was collected from the orbit of each mouse. The mice were sacrificed by cervical dislocation. The abdominal cavity was opened using surgical scissors, and the intestinal canal was dissected. Furthermore, 6 cm jejunum and ileum segments were taken. RNA was extracted from the first 1/2 segment to detect the expression of the inflammatory factor-related mRNAs. The first two segments were fixed in 10% neutral-buffered formalin. The lung tissues of mice were aseptically extracted and evenly divided into three parts. One-third of the total RNA was extracted to detect the expression of inflammatory factor-related mRNA. The other third was homogenized, and the last third was fixed in 10% neutral buffered formalin.

The lung tissue of the mice was aseptically removed, and the connective tissue was trimmed. The blood on the surface was absorbed with filter paper, and the changes in lung organ index were weighed and recorded using an analytical balance. The formulae used in this study were *A* = B/C^*^100%, where A represents the lung organ index, B represents the lung weight (mg), and C represents the body weight (g); and *D* = (E − F)/G^*^100%, where D represents the inhibition rate of the lung index (%), E represents the average lung index of the infected group, F represents the average lung index of the administration group, and G represents the average lung index of the infected group (Han et al., 2021; Song et al., 2021; Li-Juan et al., 2022; Wei et al., 2022).

### Quantitative real-time reverse transcription-PCR was used to detect the expression of related genes

The homogenate supernatants of the lung and small intestine were collected, and the amounts of virus, Interleukin-6 (IL-6), and tumor necrosis factor-α (TNF-α) in the mouse lung were detected using quantitative real-time reverse transcription PCR (qRT-PCR), as was the expression level of Interleukin-1β (IL-1β) and interleukin-10 (IL-10). After homogenization at 4°C and 12,000 RPM, centrifugation was performed for 15 min, after which the supernatant was collected, isopropanol was added at an equal volume to the supernatant, and the mixture was allowed to stand before centrifugation. The supernatant was discarded, and the precipitate remained.

In addition, 1 ml of 75% ethanol was added to each tube and mixed evenly. The supernatant was then centrifuged, discarded, and dried for 5 min. A total of 60 μl of DEPC-treated water was pipetted repeatedly until it was evenly mixed. The sample was dissolved at room temperature, shaken to properly mix, and centrifuged at a low speed for 10 s. After the system was prepared, 20 μl was centrifuged at a low speed for 10 s, vortexed, properly mixed, and then centrifuged again. The reaction parameters were 95°C for 1 min (preheating), 95°C for 15 s, and 60°C for 30 s (40 cycles), followed by the production of a dissolution curve (95°C for 15 s, 60°C for 1 min, and 95°C for 15 s) using β-actin as an internal reference gene. After the reaction, 2^−ΔΔCt^ and the relative gene expression were calculated using the CT method.

### Detection of LPS in serum by ELISA

After 30 min of blood sample agglutination and centrifugation, the serum sample was drawn and stored at −80°C. A concentrated standard sample was used for gradient dilution to prepare a standard curve of the serum sample. All the reagents and samples were maintained at room temperature before testing. After soaking the enzyme standard plate, add the standard, add the standard diluent to the blank well, and add 1 × to the sample well detection buffer 90 and 10 μl of sample. Following the kit instructions, double wavelength detection was performed using a microplate reader to determine the optical density (OD) value at the maximum absorption wavelength of 450 nm and a reference wavelength of 570 nm.

### Pathological examination

Paraffin sections of the lung and intestine were stained with H&E and alcian blue. After HE staining, the structure of small intestine wall was observed under light microscope. 10 slices of small intestine were taken from each group of mice. Each slice had 10 visual fields, and digital photography was taken. In each photo, the deepest recess depth (subject to the junction of intestinal gland villi to the base of intestinal gland), the thickest mucosal thickness and muscular layer thickness were measured. The morphology and distribution of goblet cells in the epithelium were observed under light microscope, and the number of goblet cell positive cells distributed in each recess was counted. The expression levels of zonula occludens 1 (ZO-1) and occludin were detected by immunohistochemistry. Pathological sections were randomly photographed at magnifications of 100 × and 200 ×. The OD values of the immunohistochemical images were analyzed using ImageJ software to obtain relative expression.

For immunohistochemistry, the fixed small intestine was removed, embedded in paraffin, cut into 5-μm-thick slices, and H_2_O_2_ was added and incubated at room temperature for 10 min. After rinsing, 5% bovine serum albumin (BSA) was added and incubated at room temperature for 60 min. Rabbit anti-mouse primary ZO-1 and occludin antibodies were added dropwise without washing before incubation at 4°C overnight. After rinsing, biotinylated sheep anti-rabbit IgG and rabbit anti-goat IgG antibodies were added. The sample was then washed and 3, 3′ diaminobenzidine tetrahydrochloride (DAB) was added at room temperature for 60 min.

For Alcian blue staining, the slices were placed in a drying oven and baked at 66°C for 20–30 min. Three courses of xylene and three courses of ethanol were added successively. Alcian blue staining solution (100 μl) was added dropwise to each slice, which was then dyed in a wet box for 1 h. The staining solution was removed, 100 μl of nuclear solid red staining solution was added to each slice, and sections were washed with water. These samples were passed through three successive passes of ethanol, phenol-xylene, I, and xylene II.

### Analysis and characterization of the intestinal microbiota

16S rDNA high-throughput sequencing was performed on the mouse feces collected from each group. The total DNA of bacteria in feces was extracted, and 10 ng of DNA template was used for PCR amplification according to the sequence of the v3–v4 region. The library was constructed using a library building kit, quantified using Qubit and qPCR, and sequenced on a computer. Representative sequences were selected and annotated, and the species were classified using a database.

### Western blotting

Fresh small intestinal tissue was collected, and proteins were extracted and quantified. SDS–PAGE, membrane transfer, blocking, and incubation of both primary and secondary antibodies, as well as chemiluminescence and development were carried out. The grayscale of the target protein and internal reference protein was scanned using ImageJ software, and then semiquantitative analysis of the protein content was conducted to obtain NF-κBand p-NF-κB (Phospho NF-κB) levels, as well as the relative expression of Toll-likereceptor4(TLR4) and myeloid differentiation factor 88 (MYD88).

### Network pharmacology

Chinese drug compounds were searched in the Traditional Chinese Medicine Systems Pharmacology Database and Analysis Platform (TCMSP) and Traditional Chinese Medicines Integrated Database (TCMID). The PubChem database was used to obtain the structures of the described components, which were imported into the Swiss target prediction database. A target with a prediction score greater than 0 was considered the drug target, and the Batman database was used to obtain the components and targets of gypsum. Oral bioavailability (OB) and drug-likeness (DL) were set at ≥ 30% and ≥ 0.18, respectively, in the TCMSP database to screen for effective components in the LHQW drug group. Finally, the components of Rhodiola in the TCMID database and gypsum in the Batman database were searched (Table 1).

**Table 1:** Statistics of basic information of traditional Chinese Medicine components - com–onents - targets in drug group.

Drug name	Number of components	Number of predicted targets
Radix isatidis	39	575
Mint	10	234
Chinese rhubarb	10	345
Licorice	92	777
Cyrtomium fortunei	7	278
Patchouli	11	307
Rhodiola	26	337
Honeysuckle	23	372
Semen armeniacae amarae	19	398
Forsythia suspensa	23	485
Ephedra	23	348
Gypsum	3	15
Houttuynia cordata	7	278

A total of 253 potentially active components were obtained, and 1,077 drug targets were screened. The GeneCards database was searched using “influenza virus” as the keyword. The targets were selected with an evaluation score greater than 10, and the disease targets were obtained after the removal of weightings.

Cytoscape 3.7.2 software was used to construct the “drug component target disease” network diagram, and a network analyzer was used to analyze the topology of the network diagram. In addition, a PPI cluster analysis diagram was drawn using Cytoscape software. After running the common target in the R language, gene ontology (GO) analysis identified the molecular function, cell composition, biological process, and Kyoto Encyclopedia of Gene and Genome (KEGG) pathway of the top 20 hits.

### Molecular docking

The Schrodinger software was used to construct a ligand molecular database for molecular docking. The crystal structure was downloaded from RCSB PDB. The protein structure was imported into Maestro 11.9 platform, and the protein structure was prepared using Schrodinger. The receptor was pretreated, optimized, and minimized (the OPLS3e force field was applied for constraint minimization).

### Molecular dynamics simulation

Desmond version 2020 was used for the MD simulation of proteins and compounds. OPLS3e was selected as the molecular force field for the MD simulation, and the TIP3 water model was used to solvate the system. Energy minimization of the entire system was achieved using the OPLS3e force field (all-atomic force field). A Berendsen coupling algorithm was used to create coupling between the temperature and pressure parameters. In the later preparation of the system, 100 ns were run at a time step of 1.2 femtoseconds, and the track was recorded every 10 ps, recording a total of 1,000 frames. The root-mean-square deviation (RMSD) of backbone atoms was calculated, and graphical analysis was performed to illustrate the nature of the interactions between proteins and ligands. The root-mean-square fluctuation (RMSF) of each residue was calculated to determine the major conformational changes in each residue between the initial and dynamic states.

### Calculation of MM-GBSA

The basic principle of the molecular mechanics/Poisson Boltzmann (generalized boron) surface area (MM-GBSA) method is to calculate the difference between the binding free energies of two solvated molecules in the binding and free states or to compare the free energies of different solvated conformations of the same molecule.


$$\Delta G_{\mathrm{vacuum}}^{0}=\Delta E_{MM}^{0}-T\Delta S^{0}=\left(\Delta E_{int}^{0}+\Delta E_{vdw}^{0}+\Delta E_{ele}^{0}\right)-T\Delta S^{0}$$

where $\Delta E_{int}^{0}$ includes the bond, bond angle, and dihedral angle energies, $\Delta E_{vdw}^{0}$ is the non-bond van der Waals energy, $\Delta E_{ele}^{0}$ is the nonbond electrostatic energy, and $T\Delta S^{0}$ is the entropy contribution, which can be obtained by normal mode analysis.

### Statistical processing

All statistical analyses were performed using the SPSS software (version 18.0). Measurement data are expressed as $\bar{\chi }$± s. Multiple groups were compared using one-way ANOVA. The LSD-t test was used to compare data between the groups.

## Results

### Lianhua Qingwen capsule reduces lung injury

In this study, the degree of lung injury was evaluated using the mouse lung index, lung inhibition rate, and lung histology (HE staining). Compared with the control group mice, H1N1 infected mice (infected group) showed reduced diet (Figures 1E,F) and weight loss (Figures 1C,D). The infected group had a significantly higher lung index (*p* < 0.01, Figures 1I,J), widened alveolar septum in the lung tissue, increased inflammatory cell infiltration, and partially collapsed alveolar cavities (Figure 1M). Compared with the infected group, LHQW reduced the viral load in the lungs (*p* < 0.01, Figures 1G,H) and alleviated the reduction in body weight (*p* < 0.05, *p* < 0.01, Figures 1C,D). The LHQW group had a significantly decreased lung index, reduced inflammatory cell infiltration in the pulmonary alveoli and mesenchyme, and a significantly reduced degree of pulmonary interstitial swelling (*p* < 0.01, Figures 1I–M). The survival rate of mice was significantly higher in the LHQW group (100%) than that in the infected group (72%) (Figures 1B, *p* < 0.01). Body weight change, total daily food consumption of mice, viral load in mouse lung tissue, lung index, and lung inhibition rate were separately classified and counted for female and male mice. According to the results of statistical calculation, the sex difference is not significant (*p* > 0.05), so no sex classification statistics was carried out in the subsequent experiments.

### Lianhua Qingwen capsule improves the intestinal microbe community

**Figure 2:**
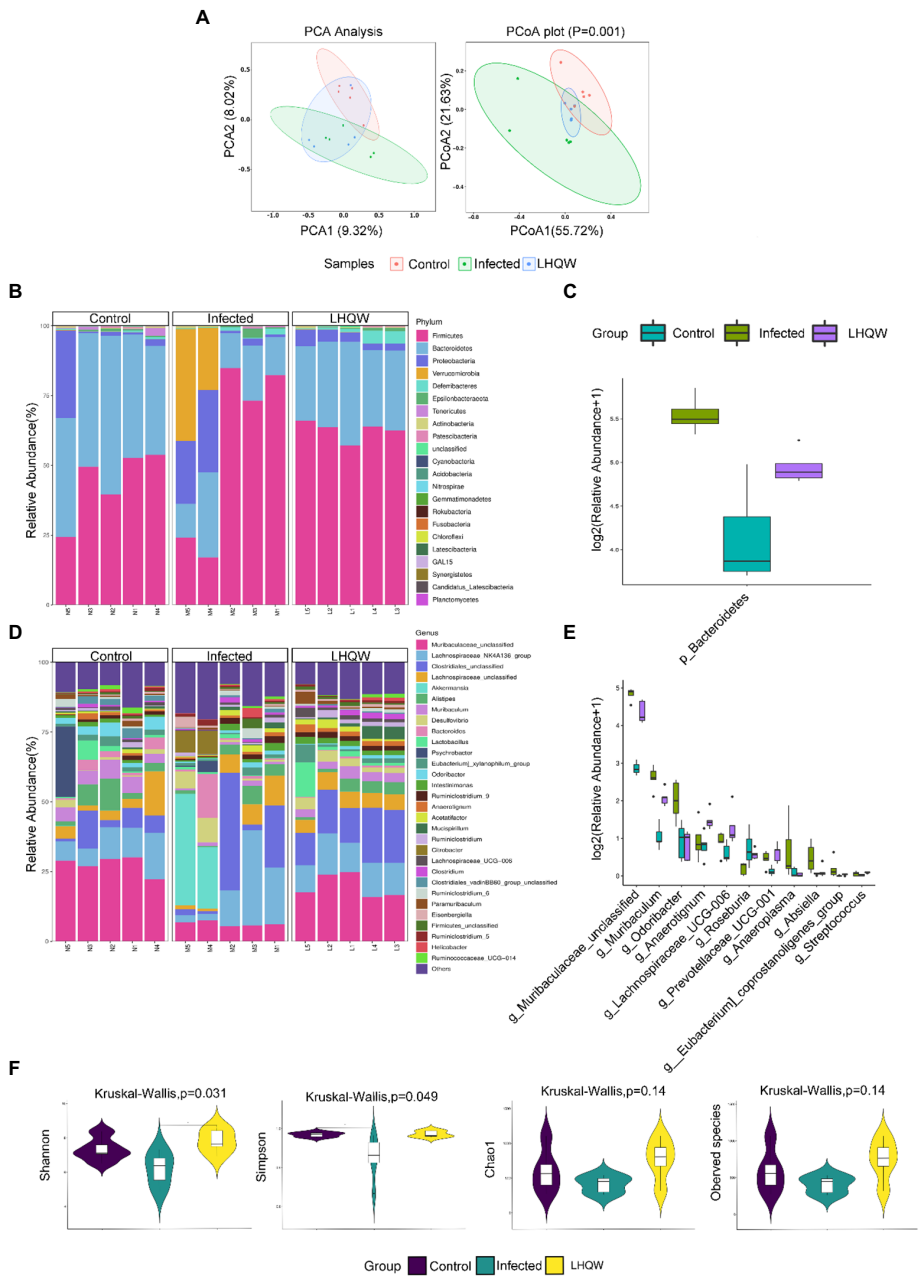
LHQW significantly improves the imbalance of intestinal flora induced by H1N1. **(A)** Beta diversity observed using principal component analysis (PCA) and principal coordinates analysis (PCoA). The same group is circled according to the 95% confidencce interval. The closer the distance between the two samples, the more similar the microbial composition structure between the samples and the smaller the difference. The sp©re analyzed at the phylum and genus levels, and those with significant differences were used to draw a box diagram. Using the genus level as an example, the abscissa represents the species with significant difference with a value of p less than 0.05 in the difference analysis, arranged from left to right according to the abundance from high to low. The ordinate represents the relative abundance. The figure shows the relative abundance of each species in each group. The taxonomic distribution of intestinal bacterial composition at the phylum level, including column stacking diagram **(B)** and box line diagram **(C)**; The taxonomic distribution of intestinal bacterial composition at the genus level, including column stacking diagram **(D)** an©ox line diagram **(E)**. **(F)** Alpha diversity indexes, including Chao1, observed species, Shannon, and Simpson indexes. Chao1 and ACE indexes are mainly used to evaluate flora richness. The larger the value, the higher the flora richnes’. Shannon and Simpson’s indexes are mainly used to evaluate flora diversity. Higher Shannon index and lower Simpson index indicate higher species diversity of the sample.

There were differences between the three groups, indicating no errors in the experimental group (Figure 2A). The stacked bar chart of the intestinal microbiota classification at the phylum and genus levels showed that the intestinal microbiota composition was significantly different (Figures 2B,D). Compared with those in the control group, the four indices of alpha diversity of the intestinal microbiota in the infected group decreased to varying degrees, of which the Simpson index decreased significantly (*p* < 0.05). At the phylum level, the microbiota abundance (*Bacteroidete*s) decreased significantly, and at the genus level, the microbiota abundance (*Muribaculaceae*_unclassified, *Muribaculum*, *Odoribacter*, *Lachnospiraceae*_ UCG-006, *Prevotellaceae*_UCG-001, *Anaeroplasma, Absiella, Eubacterium_coprostanoligenes*_Group, and *Streptococcus*) decreased significantly. Compared with the infected group intervention, LHQW reversed the red function in the four indices to varying degrees, and the Shannon index increased significantly (*p* < 0.05) (Figure 2F). The microbiota abundance (*Bacteroidetes, Muribaculaceae*_unclassified, *Muribaculum, Lachnospiraceae*_ UCG-006, and *Prevotellaceae*_Ucg-001) was significantly higher in the LHQW group and was close to that of the control group (*p* < 0.05).

### Lianhua Qingwen capsule reduces intestinal inflammation and improves intestinal mucosal barrier function

**Figure 3:**
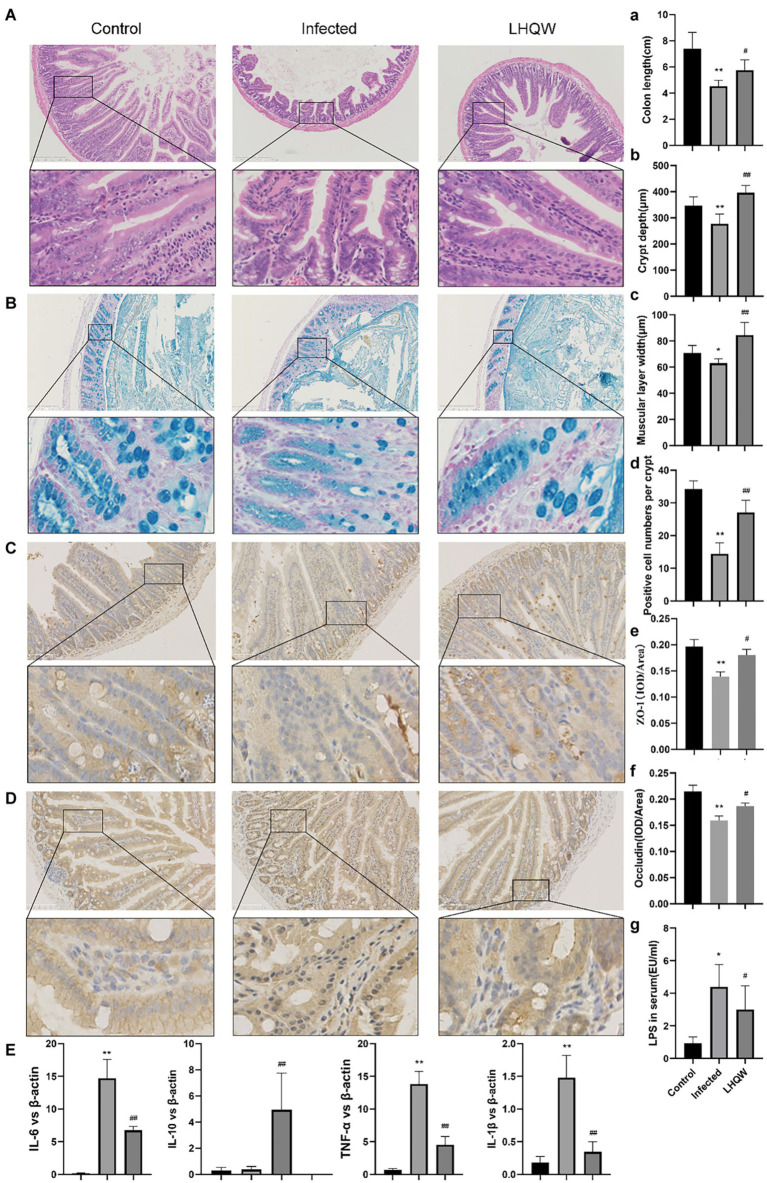
LHQW reduces intestinal wall inflammation and improves intestinal mucosal barrier function. **(A)** Small intestinal HE (100 times, 200 times) and **(B)** small intestinal recess depth **(C)** small intestinal muscle layer width according to HE; **(A)** Colon length; **(B,D)** the number of goblet cells; Immunohistochemical staining of intestinal mucosal barrier©otein **(C)** ZO-1 and **(E)** semi-quantitative analysis of Image J, **(D)** immunohistochemical staining of Occludin and **(F)** semi-quantitative analysis of ImageJ. **(G)** The content of LPS in serum of mic©as detected by ELISA. **(E)** the expression of inflammatory cytokines in the small intestinal wall was determined by qRT-PCR (IL-6, IL-1β, IL-10, and TNF-α). Compared with control group, ^*^p < 0.05, ^**^p < 0.01; Compared with infected group, ^#^*p* < 0.05, ^##^*p* < 0.01.

HE staining of the small intestine showed that the crypt depth decreased, the muscle layer thinned, and the colon length was shortened in the infected group compared to that in the control group (Figure 3A). Alcian blue staining showed that the number of small intestinal goblet cells and the amount of mucin secreted decreased in the infected group (Figure 3B). Immunohistochemical staining showed reduced expression of ZO-1 (Figure 3C) and occludin (Figure 3D) in the infected group (tan indicates positive expression; Figure 3C). In the LHQW group, the small intestinal crypt depth increased (Figure 3B), the muscle layer thickened (Figure 3C), and the colon length increased compared with that in the infected group (Figure 3A). Alcian blue staining showed that the number of small intestinal goblet cells and the amount of mucin secreted increased in the LHQW group (Figure 3D). LHQW increased ZO-1 and occludin protein expression in the intestinal epithelium. Furthermore, qRT-PCR results showed that IL-6, IL-1β, and TNF-α levels decreased, whereas IL-10 levels increased in the LHQW group compared with those in the infected group (Figure 3E).

### Network pharmacology screening results

**Figure 4:**
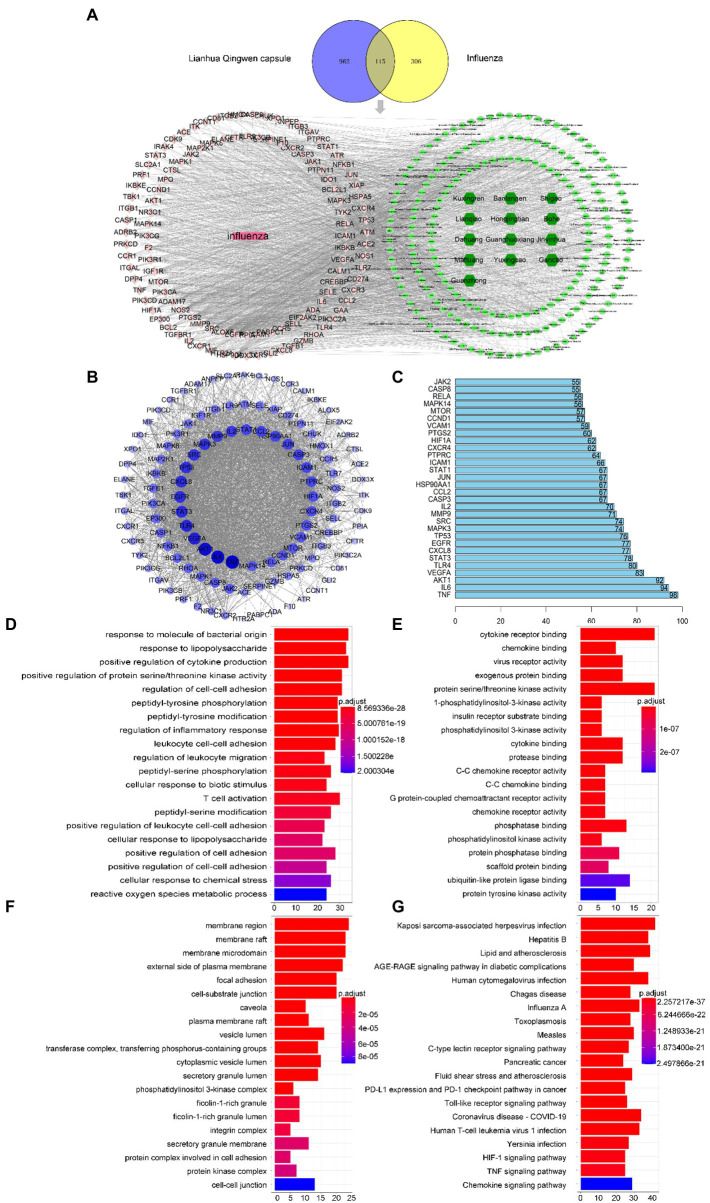
Network pharmacology screening results. **(A)** Drug disease comm“n target Wayne diagram and “d”ug component target disease” network diagram. Dark green, light green, pink, and rose red represent drugs, 204 active components in LHQW, 115 common targets, and diseases, respectively. **(B)** PPI network diagram of protein interaction. **(C)** Ranking of core targets based on PPI topology analysis (degree ranks the top 30). GO enrichment analysis of LHQW-treated influenza virus: (**©**Biological process (BP); **(E)** Cellular component **(CC)**; **(F)** Molecular function (MF). **(G)** The top 20 signaling pathways in the KEGG enrichment analysis.

Network pharmacological screening detected 1,077 drug targets and 421 disease targets. A total of 115 common drug-disease targets were obtained at the intersection of the targets (Figure 4A). The PPI network results were topologically analyzed (Figure 4B), and genes with a degree value greater than the average score were selected as the core targets (Figure 4C).

GO results showed that the intersection gene set was enriched in 2333 biological process pathways (Figure 4D), 89 cell components (Figure 4E), and 144 processes related to molecular function (Figure 4F). A total of 172 KEGG pathways were identified using R language. The top 20 terms in the KEGG enrichment bar graph (Figure 4G) were Posey’s sarcoma-associated herpesvirus infection, hepatitis B, lipids and atherosclerosis, AGE-RAGE signalling pathways in diabetes complications, human cytomegalovirus infection, Chagas disease, influenza A virus, toxoplasmosis, measles, C-type lectin receptor signalling pathway, pancreatic cancer, fluid shear stress and atherosclerosis, PD-L1 expression and PD-1 checkpoint pathway in cancer, Toll-like receptor signalling pathway, coronavirus (COVID-19), human T-cell leukemia virus 1 infection, Jerson Prand infection, HIF-1 signalling pathway, TNF signalling pathway, and chemokine signalling pathway. KEGG enrichment analysis showed that infectious diseases mainly caused hair coloring. These results indicate that the Toll-like receptor signalling pathway may be the core mechanism of LHQW-mediated amelioration of influenza A virus infection.

### Efficacy analysis of the combination mode and method

**Figure 5:**
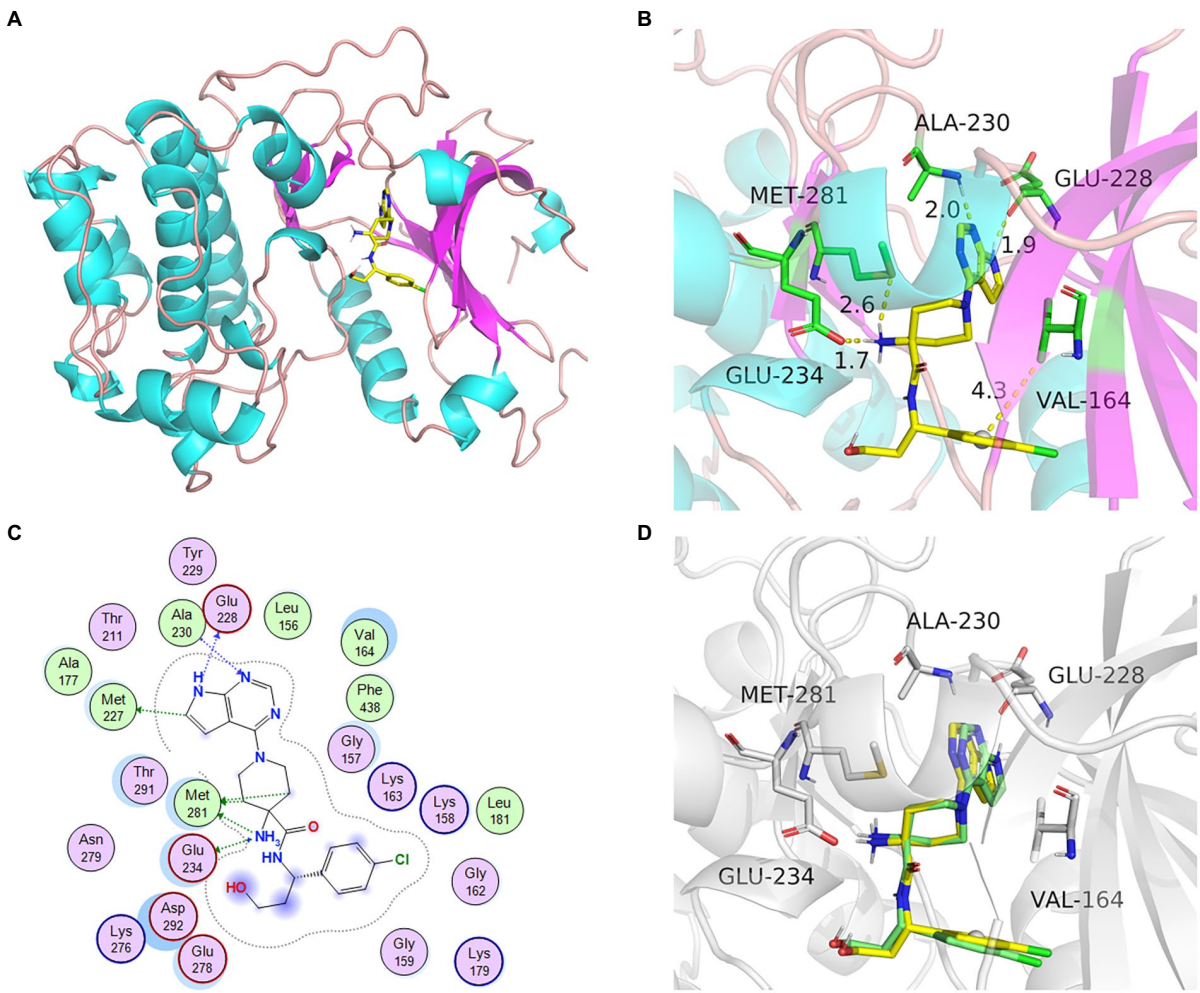
The docking model and analysis of AKT1 with natural ligand. **(A)** The overall 3D structure of AKT1 complex. The protein backbone was shown as a tube and colored bright blue and pink. The ligand was shown in stick and colored element. **(B)** A close view of the active site binding with natural ligand. Key residues interacting with ligand were shown as sticks and colored green. **(C)** The 2D protein-ligand interaction diagram of ligand-AKT1 complex. Protein residues were shown as circles and colored based on their properties: green, hydrophobic residue; purple, polar residue. **(D)** The re-docking results of active compounds with target.

AKT1 (PDB ID: 4gv1) target was used to assess the effectiveness of the docking method. The protein had high crystal structure accuracy, no deletion of key residues, clear active sites, and a clear binding mode between small-molecule ligands and active sites (Figure 5C). Protoligand molecules can form strong π-π conjugate bonds with key residues (Ala-230, Met-281, Glu-234, Val-164, and Glu-228). For instance, small ligand molecules form strong hydrophobic bonds with Val-164, thereby stabilizing small molecules. Small molecules can also form strong hydrogen bonds with amino acids (Ala-230, Met-281, Glu-234, and Glu-228) with hydrogen bond distances of 2.0, 2.6, 1.7, and 1.9 Å, respectively. They form short hydrogen bonds with strong binding ability, thus stabilizing small molecules in the active protein sites. In this study, known protoligand pairs were linked to the AKT1 binding site to determine a suitable docking method for screening potential active compounds. The binding conformation overlapped well with that of the ligands in the previous complex (Figure 5D), indicating that the screening method was suitable and effective.

### Virtual screening results

The top 50 compounds were selected based on the energy score obtained from the docking results. 10 compounds were obtained for each gene target by scoring the binding energy and evaluating the key residues of the active sites (Table 2).

**Table 2:** The docking results for AKT1, STAT3, TLR4, TNF, VEGFA, and IL6 with top 10 compound.

Target	ID	Name	Degree	Docking score (kcal/mol)
TNF	Ligand103	3-[2′-(5′-Hydroxymethyl) furyl]-1(2H)-isoquinolinone-7-O-BETA-d-glucoside	8	−11.248
	Ligand024	Licochalcone a	15	−11.209
	Ligand055	6-Prenylated eriodictyol	12	−11.091
	Ligand076	Rhein	10	−11.054
	Ligand127	(+)-Pinoresinol monomethyl ether-4-d-beta-glucoside_qt	7	−10.992
	Ligand018	Glycyrin	16	−10.898
	Ligand167	Isoglycyrol	11	−10.762
	Ligand068	Euchrenone	11	−10.751
	Ligand014	Glabrene	16	−10.728
	Ligand072	Sigmoidin-B	11	−10.319
VEGFA	Ligand073	l-SPD	11	−8.346
	Ligand169	Anthocyan	4	−7.791
	Ligand068	Euchrenone	11	−7.567
	Ligand142	Quindoline	5	−7.518
	Ligand103	3-[2′-(5′-Hydroxymethyl) furyl]-1(2H)-isoquinolinone-7-O-BETA-d-glucoside	8	−7.365
	Ligand064	DFV	11	−7.252
	Ligand100	Eriodyctiol(flavanone)	9	−7.164
	Ligand083	Eriodictyol	9	−7.138
IL-6	Ligand198	Salidroside	2	−7.086
	Ligand089	8-Prenylated eriodictyol	9	−7.046
	Ligand121	Inflacoumarin A	7	−8.586
	Ligand194	Phenanthrone	2	−7.814
	Ligand114	Machiline	8	−7.758
	Ligand093	Glycyrol	9	−7.624
	Ligand198	salidroside	2	−7.275
	Ligand153	Rutin	5	−7.212
	Ligand110	Licoricone	8	−7.124
	Ligand073	l-SPD	11	−7.082
	Ligand026	Medicarpin	15	−7.047
	Ligand104	Toralactone	8	−7.039
STAT3	Ligand113	Dinethylsecologanoside	8	−7.394
	Ligand066	Sennoside E	11	−8.992
	Ligand185	3-[[(2R,3R,5R,6S)-3,5-Dihydroxy-6-(1H-indol-3-yloxy)-4-oxooxan-2-yl]methoxy]-3-oxopropanoic acid	2	−7.098
	Ligand116	Procyanidin B-5,3′-O-gallate	7	−7.022
	Ligand173	CLR	3	−7.004
	Ligand175	Glabrone	3	−6.864
	Ligand133	Dehydroglyasperins C	6	−6.755
	Ligand164	Dehydroglyasperins C	4	−6.678
	Ligand139	(−)-(3R,8S,9R,9aS,10aS)-9-Ethenyl-8-(beta-d-glucopyranosyloxy)-2,3,9,9a,10,10a-hexahydro-5-oxo-5H,8H-pyrano[4,3-d]oxazolo[3,2-a]pyridine-3-carboxylic acid	6	−6.625
	Ligand005	Sennoside D	20	−6.624
TLR4	Ligand103	3-[2′-(5′-Hydroxymethyl) furyl]-1(2H)-isoquinolinone-7-O-BETA-d-glucoside_qt	8	−7.729
	Ligand177	Licoisoflavone B	3	−7.684
	Ligand039	7,2′,4′-Trihydroxy-5-methoxy-3-arylcoumarin	13	−7.608
	Ligand035	Vestitol	14	−7.59
	Ligand023	Kanzonols W	15	−7.59
	Ligand087	3-(2,4-Dihydroxyphenyl)-8-(1,1-dimethylprop-2-enyl)-7-hydroxy-5-methoxy-coumarin	9	−7.51
	Ligand098	Flavidin	9	−7.499
	Ligand153	Flavidin	5	−7.497
	Ligand051	Naringenin	12	−7.464
	Ligand135	Glyasperin F	6	−7.365
AKT1	Ligand063	Herbacetin	12	−8.529
	Ligand198	Salidroside	2	−8.447
	Ligand043	Phaseolinisoflavan	13	−8.327
	Ligand035	1,3-Dihydroxy-9-methoxy-6-benzofurano[3,2-c]chromenone	5	−8.275
	Ligand023	Kanzonols W	15	−8.275
	Ligand149	Glyzaglabrin	5	−8.206
	Ligand046	Kaempferol	13	−8.115
	Ligand009	Herbacetin	18	−8.115
	ligand177	Licoisoflavone B	3	−8.099
	Ligand191	Semilicoisoflavone B	2	−8.072

### Interaction analysis of small protein molecules

The core active components (204) of LHQW were docked with AKT1, STAT3, TLR4, TNF, VEGFA, and IL6 targets. The PyMOL21 software was used to visualize the complexes formed by docking. For each target, the compound was selected with the best score for protein targeting and docking (ligand063, ligand113, ligand103, ligand073, and ligand121). The binding mode between the compounds and proteins was then used to visualize the amino acid residues between the compounds and protein pockets.

**Figure 6:**
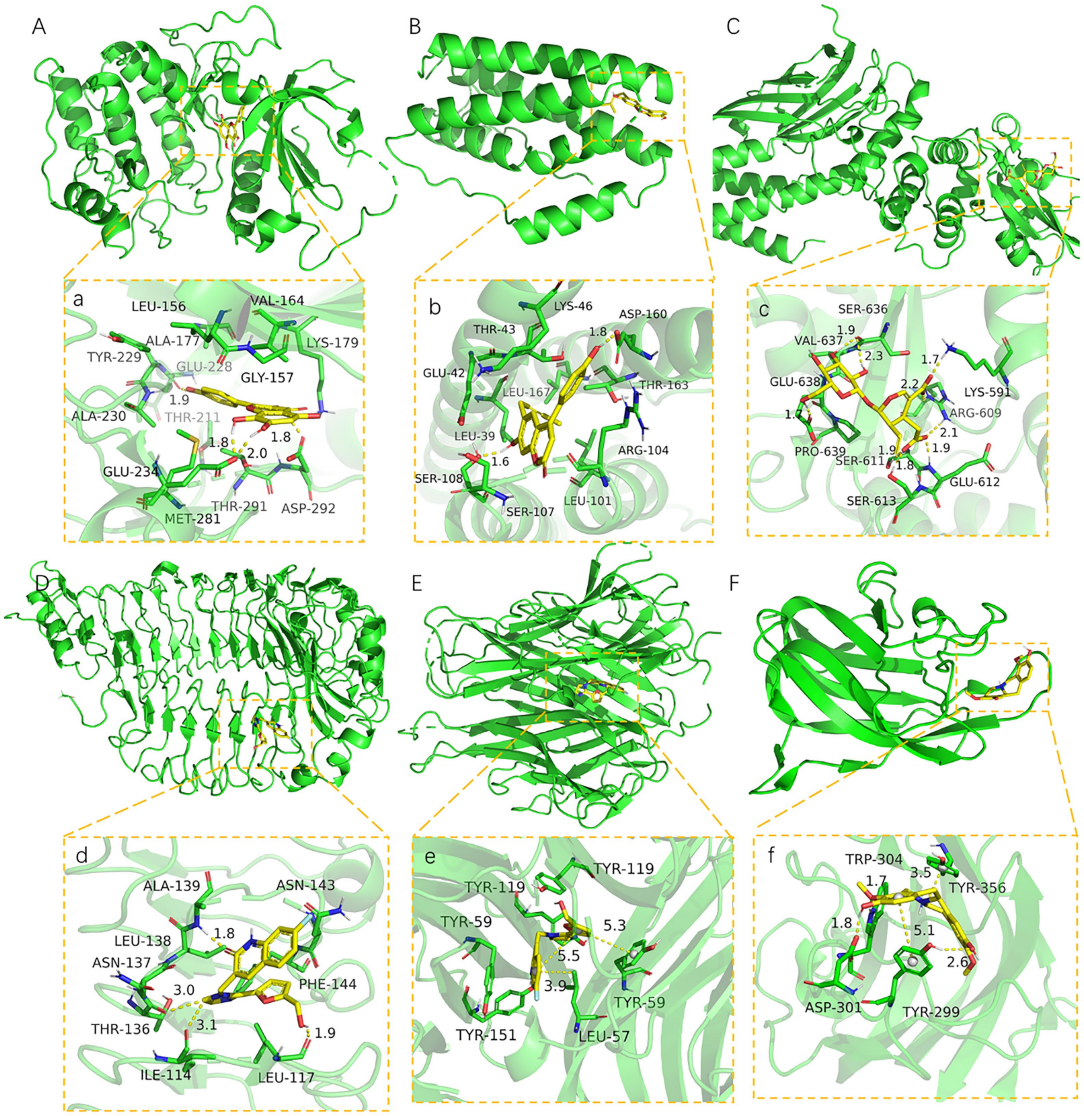
The binding site of target with compound **(A-F)**. The 3D structure of AKT1-ligand063, IL6-ligand121, STAT3-ligand113, TLR4-ligand103, TNF-ligand103, VEGFA-ligand073. **(a-f)** A close view of the active site binding with AKT1-ligand063, IL6-ligand121, STAT3-ligand113, TLR4-ligand103, TNF-ligand103, VEGFA-ligand073. Key residues interacting with ligands were shown as sticks and colored green. The protein backbone was shown as a tube and colored bright blue.

Ligand063 has multiple hydrogen bond donors and receptors and thus forms strong hydrogen and hydrophobic bonds with protein active sites (Figure 6A). The hydrogen bond distance was significantly shorter (average of 1.86 Å) than that of the traditional hydrogen bond (3.5 Å). Moreover, the ligand has a strong binding ability and thus anchors small molecules in the protein pockets. Ligand121 contains three benzene rings with strong hydrophobicity and forms strong hydrogen and hydrophobic bonds with the hydrophobic cavity of the protein (Figure 6B). The hydrogen bond distances are 1.6 Å and 1.8 Å. Ligand 113 had a six-membered sugar ring at one end and an oxygen-containing six-membered ring with multiple carboxyl groups at the other end, yielding multiple hydrogen bonds for the receptor (Figure 6C). Ligand103 and ligand073 highly matched the target protein pocket and formed hydrogen, hydrophobic, and other bonds with the protein pocket (Figures 6D-F). These results indicate that ligand063, ligand121, ligand113, and ligand073 are potential active compounds with good binding affinity and matching with AKT1, IL6, STAT3, TLR4, TNF, and VEGFA.

### Molecular dynamics simulation analysis

**Figure 7:**
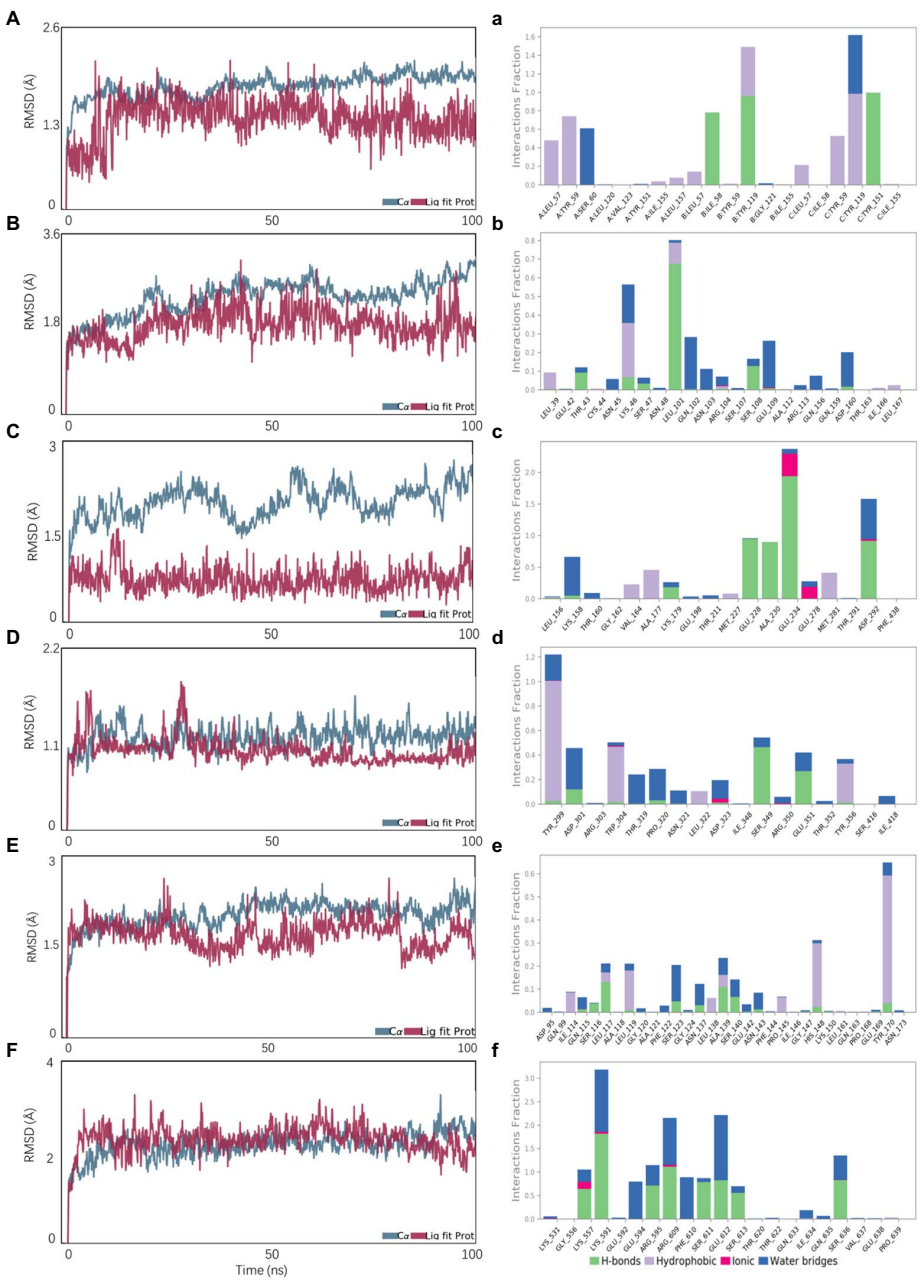
Molecular dynamics simulations. **A, B, C, D, E, F** represent the RMSD plot during molecular dynamics simulations of AKT1-ligand063, IL6-ligand121, STAT3-ligand113, TLR4-ligand103, TNF-ligand103, VEGFA-ligand073; **a, b, c, d, e, f** represent the interaction residues of AKT1-ligand063, IL6-ligand121, STAT3-ligand113, TLR4-ligand103, TNF-ligand103, VEGFA-ligand073.

The stability of reactive proteins and small molecules depends on the RMSD. The larger the RMSD, the more unstable is the protein. The stability of small molecules fluctuated at the beginning and tended to stabilize during movement, reflecting the continuous collision between small molecules and active sites in the protein pocket (Figure 7). This result also showed that the small molecules combined well with the protein pocket and reached a dynamic equilibrium. The average RMSD values of the VEGFA-ligand073, TLR4-ligand103, AKT1-ligand063, and IL6-ligand121 complexes were approximately 2, 5, 10, and 40 ns, respectively. The average RMSD value of TNF-ligand103 was < 2.4 Å and had a slight RMSD fluctuation; thus, the small molecule–protein complex could quickly reach a stable state, reflecting the good stability of the complex. The above results also indicated that the small molecule had a high degree of matching with the protein. The average RMSD of the STAT3-ligand113 complex was < 4.0 Å and showed a slight RMSD fluctuation, possibly due to the unstable chain of the protein. Protein and small molecule complexes quickly reached dynamic equilibrium, with a consistent change trend in the RMSDs of the complexes, indicating that small molecules and proteins can form stable complexes, which change with the conformational state of the proteins in the solvent. Ligand063, ligand121, ligand103, ligand073, and ligand063 formed strong hydrogen and hydrophobic bonds with the active site (Figure 7). In addition, ligand121 exhibited a strong electrostatic interaction with the active site. Ligand 113 formed a strong hydrogen bond with the active site, which induced its binding to the protein. These interactions can improve the stability of small molecules in the protein pockets.

### Results of the calculation of free binding energy

The calculation of static molecular docking and molecular mechanics generalized Born surface area (MM-GBSA) free binding energy ensures that the complexes of compounds and targets have sufficient energy for biochemical reactions by providing binding posture and binding free energy. The binding free energy calculated using MM-GBSA supported the molecular docking results. Ligand103 had the highest binding ability to TNF (210.26 ± 22.01) and TLR4 (180.53 ± 19.85; Table 3).

**Table 3:** MM/GBSA of the best candidate compounds and protein targets for each target.

Chemical compound	Target	Van der Waals force (kJ/mol)	Electrostatic potential energy (kJ/mol)	Polar solvation energy (kJ/mol)	Surface solvation (kJ/mol)	Binding free energy (kJ/mol)
Ligand063	AKT1	−258.71 ± 16.21	−89.23 ± 13.49	220.17 ± 18.22	−25.37 ± 1.46	−178.23 ± 18.29
Ligand121	IL6	−229.17 ± 13.98	−101.37 ± 17.21	232.01 ± 16.31	−30.19 ± 1.01	−156.91 ± 17.35
Ligand113	STAT3	−252.66 ± 18.21	−92.37 ± 15.76	219.32 ± 19.06	−28.37 ± 0.92	−176.22 ± 16.47
Ligand103	TLR4	−210.37 ± 12.90	−99.28 ± 16.01	211.39 ± 17.88	−33.51 ± 1.33	−180.53 ± 19.85
Ligand103	TNF	−199.58 ± 17.26	−70.56 ± 12.99	245.62 ± 15.90	−32.02 ± 1.47	−210.26 ± 22.01
Ligand073	VEGFA	−241.11 ± 11.08	−118.34 ± 13.37	260.79 ± 15.37	−27.63 ± 0.89	−128.83 ± 19.37

### Correlation analysis

Network pharmacological screening and molecular docking results showed that the TNF and Toll receptor signalling pathways targeting TNF and TLR4 might be the main pathways involved in LHQW-mediated amelioration of influenza A virus infection. The 16S rDNA results were combined with the results of molecular biology experiments (qRT–PCR and WB results of lung tissue) to determine the relationship between the amount of virus in the mouse lung and the main target of LHQW in the treatment of influenza A virus and abundance of the intestinal microbiota. The correlation between the abundance of the intestinal microbiota at the phylum and genus levels and the targets screened using computer-aided design technology was also assessed.

**Figure 8:**
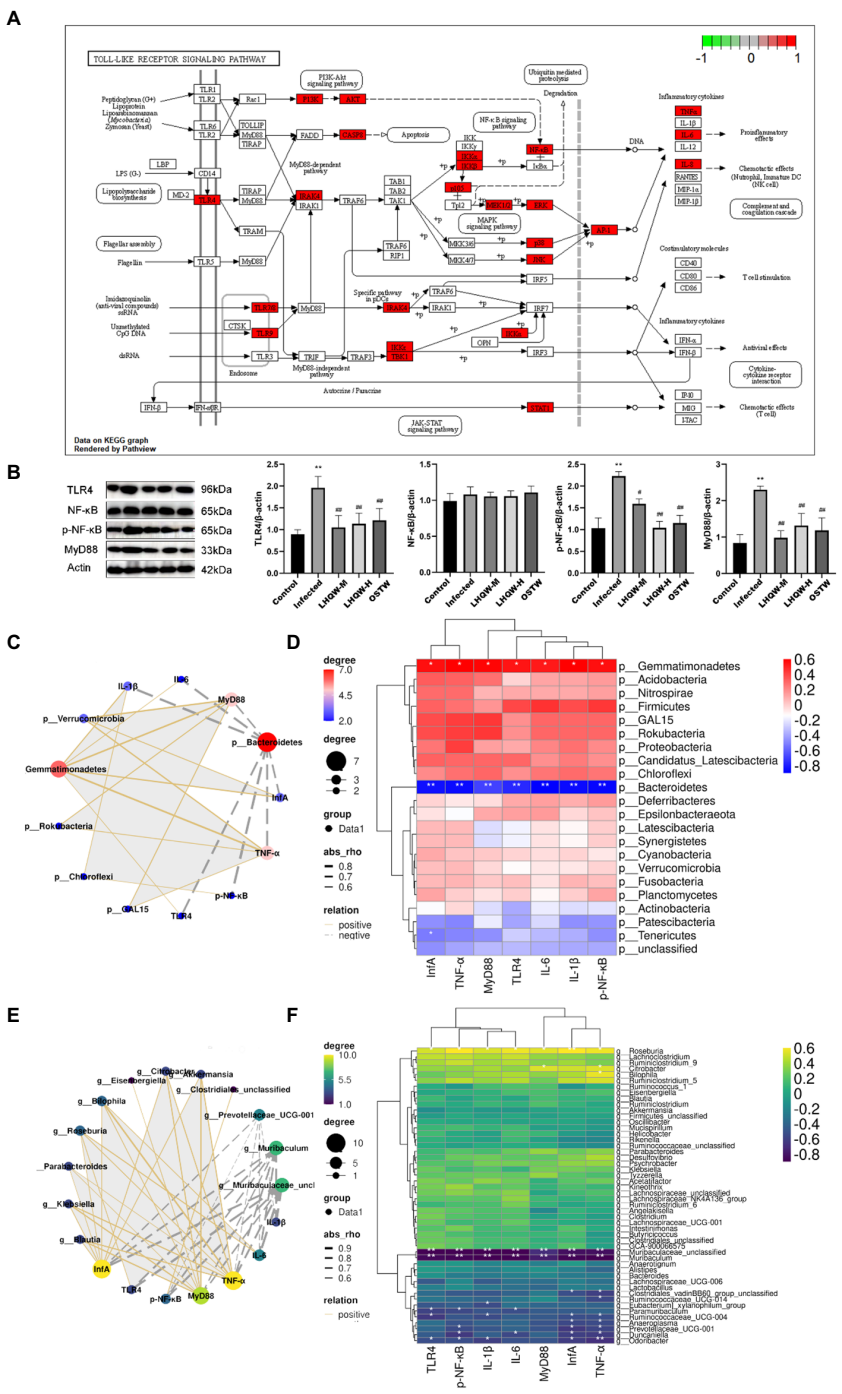
Correlation analysis. **(A)** Toll-like receptor signaling pathway; **(B)** TLR4, NF-κB, and p-NF-κB levels in mouse lung tissue. MyD88 protein expression level; Correlation network diagram **(C)** and heat diagram **(D)** at the phylum level; Correlation network diagram **(E)** and heat map **(F)** at the genus level. Compared with control group, ^*^p < 0.05, ^**^p < 0.01; Compared with infected group, ^#^*p* < 0.05, ^##^*p* < 0.01.

At the phylum level, the influenza A virus (Inf A) and TLR4, NF-κB, TNF-α, IL-6, and IL-1β levels were negatively correlated with *Bacteroidetes* abundance. In contrast, these levels were positively correlated with *Verrucomicrobia*, *Gemmatimonadetes*, Rokubacteria, and *Chloroflexi*. At the genus level, INF-α, TLR4, NF-κB, TNF-α, IL-6, and IL-1β levels were negatively correlated with Prevotellaceae_UCG-001, *Muribaculum*, and Muribaculaceae_unclassified abundances. In contrast, these levels were positively correlated with *Blautia*, *Klebsiella*, *Parabacteroides*, *Roseburia*, *Bilophila*, *Eisenbergiella*, *Citrobacter*, Akkermansia, and *Clostridiales*_unclassified abundance (Figure 8).

## Discussion

Oseltamivir (OSTW), a neuraminidase inhibitor commonly used to treat influenza, is an antiviral drug listed in China. OSTW is a classic antiviral drug with good antiviral effects. However, based on the principle of neuraminidase inhibition, the protective effect of this drug on influenza patients has a time window. Taking it 48 h after onset can significantly shorten the course of the disease and inhibit virus replication. Therefore, we selected OSTW as the positive control drug in this study. BALB/c inbred line mice had the same genetic background. The use of Balb/c mice can reduce the influence of genetic background factors among individual mice on the difference in virus infection sensitivity; therefore, it is easier to study and analyze the dynamic characteristics of viral pathogenicity and replication. BALB/c mice were selected for this study according to the standard operating procedures of the Institute for Viral Disease Control and Prevention (2007).

In this study, we found that the inflammatory factors in the lungs of mice with viral pneumonia were disordered, the lungs were damaged, and the TLR4 pathway was significantly activated (Figure 8B). One of the important factors of the influenza virus leading to human death is the abundant expression of pro-inflammatory cytokines. Some studies have shown that lung damage caused by influenza A virus (IVA) is mainly caused by an excessive inflammatory response rather than direct damage to the alveolar epithelium by the virus (Brandes et al., 2013). Excessive immune response can cause a “cytokine storm” that induce acute lung injury (ALI), and further develop acute respiratory distress syndrome (ARDS), leading to respiratory failure and even death. Therefore, in the treatment of influenza virus infection, it is very important to reduce the inflammatory response caused by IVA in the lung (de Jong et al., 2006; Perrone et al., 2008; Bermejo-Martin et al., 2009; Brandes et al., 2013; Li et al., 2014).

During the course of influenza virus infection, three different types of innate immune pattern recognition receptors (PRRs) recognize viral RNA, namely Toll-like receptors (TLRs), retinotide-induced gene I (RIG-I), and NLRP3. After receptor activation, interferon regulators 3/7 (IRF 3/7) and NF-κB are activated to promote the expression of IFNs and pro-inflammatory factors, respectively (Sun et al., 2010). TLRs are the main PRRs for host virus recognition, and during IVA infection, TLR 3/7 is involved in pathogen-associated molecular pattern (PAMPs) identification from IVA by activating myeloid differentiation factor 88 (MYD88), activating downstream transcription factors NF-κB or IRF7, and promoting the expression of pro-inflammatory cytokines such as IL-lβ, IL-18, and type I IFN, respectively (McKinstry et al., 2009).

TNF-α is considered the major pro-inflammatory cytokine capable of causing “cytokine storm” (Cheung et al., 2002), thereby exacerbating the pathogenicity of influenza viruses in humans. (Peper and Van Campen, 1995) found that inhibition of TNF-α reduced lung inflammation but did not affect the clearance of the influenza virus. IL-6 plays a crucial role in resistance to H1N1 influenza infection by promoting neutrophil survival (Dienz et al., 2012). A large body of evidence has shown that the presence of IL-6 may be a marker of persistent inflammation and a direct participant in a comprehensive immune response (Dienz et al., 2012; Spencer et al., 2019; Yousif et al., 2021), IL-6 levels in patients with acute exacerbation were significantly increased compared with those in the stable stage and were positively correlated with viral infection (Hutchinson et al., 2010). IL-1β may be a key inflammatory factor that leads to acute lung injury and ARDS (Fukuyama and Kawaoka, 2011; Niu et al., 2019). It is produced by monocyte-macrophages and endothelial cells and is a pro-inflammatory cytokine involved in host defense responses, such as the immune response to inflammation. It can activate vascular endothelial cells and inflammatory cells such as T lymphocytes and macrophages, release inflammatory mediators, and promote effector cells of the immune system to enter the infection site (Yazdi and Ghoreschi, 2016).

The results of this study showed that the alpha diversity of the intestinal microbiota, the abundance of *Bacteroidetes, muribaculaceae*_unclassified, and *Streptococcus* decreased significantly (Figure 2), accompanied by disordered intestinal wall inflammatory factors and injured intestinal mucosal barriers (Figure 3). LHQW treatment increased the survival rate, restored the alpha diversity of intestinal microbiota, recovered the *Bacteroidetes* and *muribaculaceae*_unclassified levels, adjusted inflammatory factors of the intestinal walls, and improved the intestinal mucosal barrier (Figure 2).

Intestinal microbiota-derived propionic acid protects against zinc oxide nanoparticle-induced lung injury (Zhang Y. et al., 2022). Baicalin inhibits APEC-induced lung injury by regulating gut microbiota and short-chain fatty acids (SCFA) production (Peng et al., 2021). Mycoplasma gallisepticum baicalin ameliorated-induced inflammatory injury in the chicken lung by regulating the intestinal microbiota and phenylalanine metabolism (Wang J. et al., 2021). The lung and gut microbiota are altered by hyperoxia and contribute to oxygen-induced lung injury in mice (Ashley et al., 2020). Gallacher et al. (2020) found that targeted treatment of the predominant organisms, including those not routinely treated, such as spp., may decrease the development of CLD in preterm-born infants. In summary, these findings suggest that restoration of normal intestinal microbiota can repair lung injury. The lung and intestine have a similar mucosal immune system, which is functionally interconnected. Influenza virus can not only cause lung inflammation but can also be accompanied by gastrointestinal symptoms such as diarrhea and vomiting after infection with influenza H7N9 virus (Gao et al., 2013). In this experiment, we observed numerous inflammatory cell infiltrations in the lungs of mice with pneumonia and significantly increased expression of TNF-α, IL-1β, and IL-6 in the lungs, accompanied by intestinal wall barrier damage, as well as significant changes in the intestinal microbiota (Figures 1C, 2, 3, 8). These phenomena suggest that the pulmonary inflammatory response caused by the virus may lead to cytokines or immune cells entering the intestinal wall through the blood circulation, causing an immune response and resulting in damage to the intestinal wall barrier. Wang et al. (2014) reported that after mice were infected with H1N1 intranasally, the CCR9^+^ CD4^+^ T cells in the lungs migrated to the small intestine mediated by CCL25/CCR9 and secreted IFN-γ, changing the composition of the intestinal microbiota and leading to an increase in IL-15 secretion by intestinal epithelial cells, thereby promoting *in situ* differentiation of CD4^+^ T cells into Th 17 cells, which eventually triggered intestinal inflammation. Other studies found that in BALB/ C mice inoculated intranasally with influenza H1N1 virus, a significant inflammatory response was observed in the large and small intestine, accompanied by intestinal micromicrobiota disorder (Zhang et al., 2015; Li et al., 2018). Another study reported that after influenza virus infection in an animal model, the abundance of intestinal microbiota, such as *Bifidobacterium lactobacillus*, was significantly reduced, *Enterococcus* and *Enterobacteria* increased significantly, and the bacteria associated with the production of short-chain fatty acids (SCFAs) decreased significantly, which plays an important role in intestinal defense against pathogenic bacteria (Wang et al., 2014; Adak and Khan, 2019).

*Bacteroidetes, Muribaculaceae,* and *Streptococcus* are bacteria that produce SCFCs. The intestine has several lines of defense that protect it against the translocation of microorganisms or microbial products into the bloodstream. This multilayer barrier constitutes the largest interface between the external environment and host (Zhang et al., 2021). The gut-vascular barrier, an additional cellular barrier, is situated below the epithelial barrier and is involved in controlling the translocation of microorganisms into the portal vein (Buckley and Turner, 2018). In addition, gut permeability can be altered by gut microbiota dysbiosis, favoring LPS translocation into systemic circulation, with the development of low-grade endotoxemia. LPS is a component of the membrane of gram-negative bacteria present in the gut that can translocate into the systemic circulation, causing non-septic, low-grade endotoxemia (Violi et al., 2022). Gut dysbiosis is a major determinant of low-grade endotoxemia *via* dysfunction of the intestinal barrier scaffold, which is a prerequisite for LPS translocation into systemic circulation (Johansson et al., 2013; Arbizu et al., 2020). LPS is widely used as an important indicator of intestinal permeability (Li et al., 2021; Liu et al., 2021; Wang Y. et al., 2021; Zhao et al., 2021). Therefore, the detection of LPS in serum by ELISA can be used to evaluate intestinal permeability. In this experiment, the diversity of the intestinal microbiota and the abundance of *Bacteroides*, *Muribaculaceae,* and *Streptococcus* were significantly reduced, resulting in an increase in the abundance of opportunistic bacteria (Figure 2), which then led to intestinal mucosal barrier damage. In this case, enterobacteria metabolites, such as LPS, enter the damaged intestinal wall, aggravate the intestinal wall immune response, and release inflammatory factors, and then amplify the intestinal wall injury, which crosses the injured intestinal wall into the bloodstream and activates the TLR4 signalling pathway in the lungs to aggravate the inflammatory response and injury in the lung, which was also verified in our experiments.

The results of this study showed that LHQW treatment reduced the viral loads and adjusted inflammatory factors in the lungs, alleviated lung injury, and inhibited the activation of the TLR4/NF-κB signalling pathway in viral pneumonia mice (Figures 1C, 8B). Network pharmacological analysis showed that the six active herbal medicine molecules from LHQW could regulate the intestinal microbiota, thereby inhibiting the immune-inflammatory response through the TLR4/NF-κB signalling pathways in the lungs (Table 2). It reveals the relationship between the gut microbiota, intestinal mucosal barrier, and viral pneumonia, and part of the mechanisms of LHQW in the treatment of viral pneumonia. The LHQW capsule, a traditional Chinese medicine compound, has 13 traditional Chinese medicines and many action targets, such as Akt1, P53, Cox-2, and TLR4 (Xia et al., 2020; Su et al., 2022), and has unique advantages in the treatment of influenza virus infection. Formula composition: forsythia suspensa 225 g, honeysuckle 255 g, isatis root 255 g, bitter almond 85 g, menthol 7.5 g, houttuynia cordata 255 g, rhubarb 51 g, patchouli 85 g, Male Fern Rhizome 255 g, Rhodiolae Herba 85 g, Ephedra equisetifolia 85 g, licorice 85 g, gypsum 255 g. Studies have confirmed that the main active ingredients of LHQW are chlorogenic acid, ursolic acid, rutin, luteolin, and quercetin, which exhibit antibacterial activities (Niu et al., 2021; Yang et al., 2021; Zhou et al., 2021).

In this experiment, after treatment with LHQW, the alpha diversity and abundance of probiotics such as *Bacteroidetes, Muribaculaceae,* and *Streptococcus* in the intestinal tract of mice with viral pneumonia were significantly increased, and intestinal mucosal barrier injury was significantly alleviated (Figures 2, 3).

**Figure 9:**
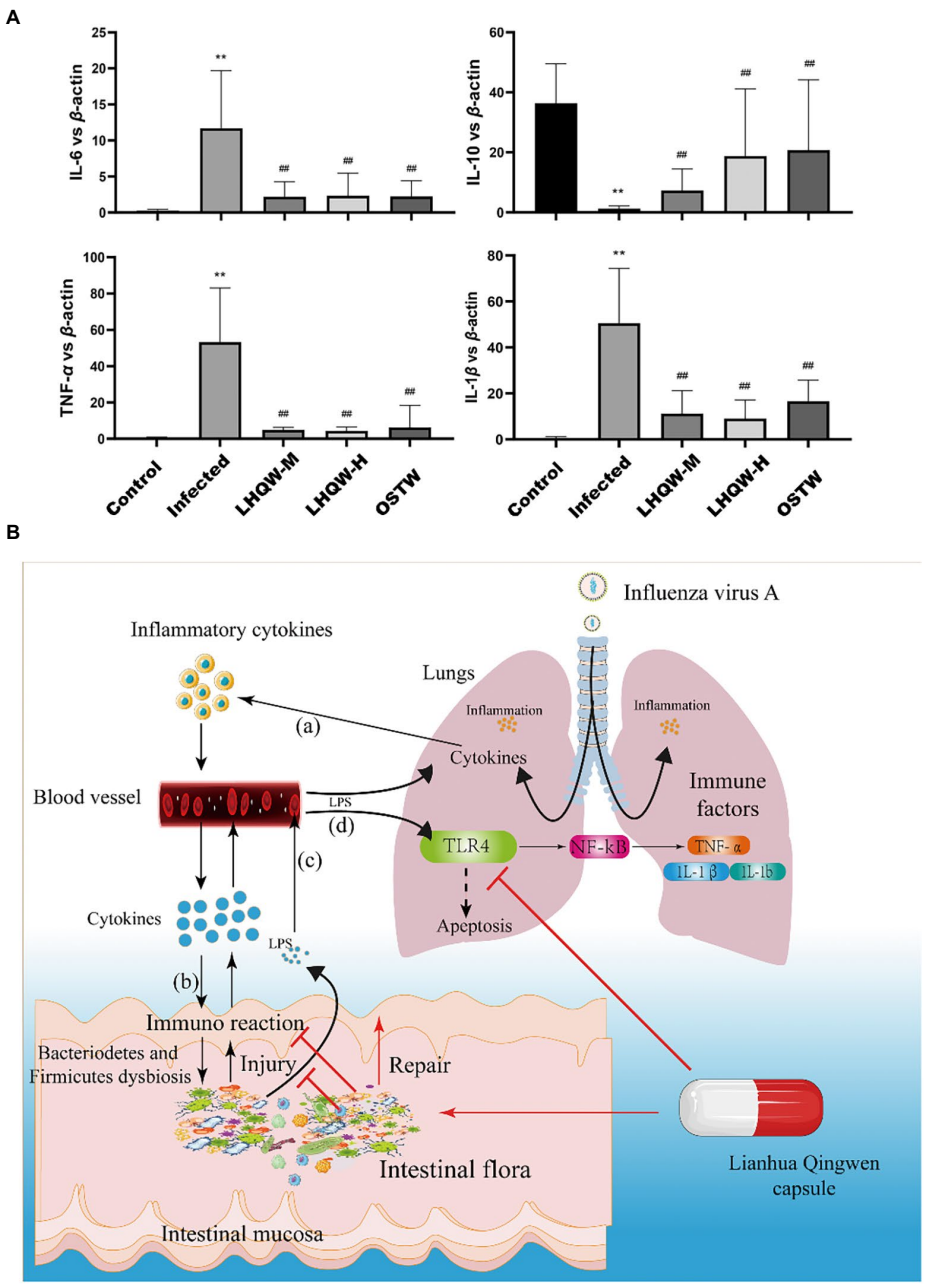
**(A)** qRT-PCR detection of inflammatory factors (IL-6, IL-1β, IL-10, TNF-α) in the mouse lung tissue. Compared with control group, ^*^p < 0.05, ^**^p < 0.01; Compared with infected group, ^#^*p* < 0.05, ^##^*p* < 0.01. **(B)** The working model of LHQW capsule against influenza A virus infection. (a) The lung infected with influenza A virus induces an immune response in lung, leading to the release of inflammatory cytokines into blood. (b) The inflammatory cytokines from the blood enter the intestinal wall to induce the immune response, resulting in intestinal wall injury. (c) The LPS from the intestine crosses the damaged intestinal mucosal barrier and enters the blood to activate the TLR4-mediated pathway in lung, causing the lung injury (d).

As a result, bacterial metabolites (LPS) entering the intestinal wall are reduced and the resulting immune response and inflammatory factors are also decreased. Meanwhile, LPS crossing the intestinal wall to enter the blood stream and eventually reach the lungs was significantly reduced, thus reducing the activation of the LPS/TLR4/NF-κB signalling pathway (Figures 8A,B), thereby decreasing the expression of inflammatory factors in the lungs. In addition, LPS/TLR4 can also be inhibited (Figure 8A), thus reducing lung injury (Figure 9), which is beneficial for the survival of some immune cells and the clearance of the virus in the lungs.

## Conclusion

The present study was designed to determine the effect of intestinal microbiota in the improvement of LHQW activity in viral pneumonia and its possible mechanism. The findings clearly indicate that LHQW is effective for treating influenza A virus infectious pneumonia, and the mechanism is associated with the regulation of the TLR4/NF-κB signalling pathway in the lungs by restoring intestinal microbiota and repairing the intestinal wall. This result provides theoretical support for the effective use of LHQW in the treatment of influenza A virus.

## Data availability statement

The data presented in the study are deposited in the NCBI repository, accession number PRJNA889462.

## Ethics statement

The animal study was reviewed and approved by all animal experimental procedures strictly followed the protocol approved by the Ethics Committee of Wannan Medical College (YJS-2020-10-006).

## Author contributions

PX, ZY, and SD performed the experiments, the data, and wrote the manuscript. ZH and SZ contributed to the study design and overall supervision. All authors have reviewed the manuscript. All authors contributed to the article and approved the submitted version.

## Funding

This work was financially supported by the Natural science projects in colleges and universities in Anhui Province (grant number KJ2020ZD56) and the National Natural Science Foundation of China (grant number 81671318). SZ received funding from the Natural science projects in colleges and universities in Anhui Province (grant number KJ2020ZD56). ZH received funding from the National Natural Science Foundation of China (grant number 81671318).

## Conflict of interest

The authors declare that the research was conducted in the absence of any commercial or financial relationships that could be construed as a potential conflict of interest.

## Publisher’s note

All claims expressed in this article are solely those of the authors and do not necessarily represent those of their affiliated organizations, or those of the publisher, the editors and the reviewers. Any product that may be evaluated in this article, or claim that may be made by its manufacturer, is not guaranteed or endorsed by the publisher.
